# A Comprehensive Analysis of the Management of Brain Metastases—Experience from a South-Eastern European Neurosurgical Centre

**DOI:** 10.3390/medicina61101773

**Published:** 2025-10-01

**Authors:** Florin Adrian Tofan, Ahmed T. Massoud, Cosmin Ioan Faur, Ioan Ștefan Florian

**Affiliations:** 1 Department of Neurosurgery, Faculty of Medicine, University of Medicine and Pharmacy Iuliu Hatieganu, 400012 Cluj Napoca, Romania; 2 Faculty of Medicine, Fayoum University, Fayoum 63514, Egypt; 3 Regina Maria Dental Department, Regina Marina Private Healthcare Network, 014416 Bucharest, Romania

**Keywords:** brain metastasis, surgical outcomes, gross total resection, prognostic factors

## Abstract

*Background:* Brain metastases represent the most common intracranial tumours in cancer patients, with no consensus on surgical outcomes and prognostic factors. This study aimed to analyse the demographic, clinical, and tumour-related factors influencing postoperative complications, recurrence, and functional outcomes in patients undergoing surgical resection of brain metastases at a tertiary neurosurgical centre in South-Eastern Europe. *Materials and Methods:* A retrospective cohort of adult patients who underwent gross total (GTR) or subtotal resection (STR) for brain metastases was analysed, stratified by sex, extent of resection (GTR vs. STR), and recurrence status. *Results:* A total of 358 patients underwent surgical resection for brain metastases, with pulmonary carcinoma being the most common primary tumour (46.4%). Most patients had solitary metastases (87.4%), and eloquent brain regions were involved in 53.9% of cases, 20.1% experienced postoperative complications, and recurrence occurred in 10.9%. Higher preoperative KPS predicted fewer complications. GTR was not associated with complications or recurrence but was linked to lower postoperative mortality. Median overall survival was 325 days. For the three hundred fifty-four patients with survival data, median overall survival (OS) was 325 days (95% CI: 270–380). OS did not meaningfully differ by sex. *Conclusions:* Gross total resection was not independently associated with reduced postoperative complications or recurrence but was significantly associated with lower postoperative mortality. Functional status, eloquent brain region involvement, and age remained key determinants of clinical outcomes.

## 1. Introduction

Brain metastases are the most prevalent intracranial tumours and a common complication of systemic cancers, with an estimated incidence of intracranial metastases in 10% to 26% of patients with malignancy [1]. The distribution of brain metastases often parallels regional cerebral blood flow; however, certain tumour types prefer certain brain territories [2]. The diagnosis of brain metastasis rapidly changes the course of management for cancers. Given the improvements in systemic therapies providing prolonged survival for many types of cancer, the detection time and treatment of intracranial disease are becoming more important [3]. New neurological signs or symptoms, seizures, or cognitive changes in any cancer patient should routinely raise suspicion of brain metastasis [4]. Treatment is multidisciplinary and is often influenced by the number of lesions, the burden of symptoms, the burden of systemic disease, and remaining functional status [5]. Surgical resection should be indicated in patients with solitary or symptomatic lesion(s), causing a mass effect [5,6]. Gross total resection (GTR) is associated with improved local control and functional outcomes. Still, the risks of the surgery, particularly related to tumour location in the eloquent cortex, and comorbidities should be involved in surgical planning [7]. While much research has been conducted on surgical outcomes, most of the data have come from North American or Western European Institutions [8,9]. Little of the literature represents practices from different global sites.

This study helps fill that gap with a thorough, single-centre experience from a neurosurgery service in South-Eastern Europe. The primary objectives of this study are to describe the demographic, clinical and tumour-related characteristics of patients treated surgically for brain metastasis. In addition, this study aimed to characterise predictors of postoperative complications, recurrence, functional outcomes, and overall survival. To our knowledge, this is the largest single-centre cohort of surgically treated brain metastases reported from South-Eastern Europe, and it integrates clinical, functional, and anatomical predictors with propensity score-based sensitivity analysis, providing insights from an underrepresented region.

## 2. Materials and Methods

### 2.1. Study Design and Setting

This retrospective cohort study was conducted at the Neurosurgery Department of the Cluj County Emergency Clinical Hospital, a tertiary neurosurgical centre in South-Eastern Europe. This study was conducted in accordance with the Declaration of Helsinki and included all adult patients who underwent surgical resection for brain metastases between January 2012 and December 2024. All analyses were confirmed via official correspondence with the Cluj County Population Records Directorate (letter ID: 16302, dated 27 May 2025).

### 2.2. Participants

Patients were eligible if they:

- Were aged 18 years or older;
- Had a histologically confirmed brain metastasis;
- Underwent surgical resection (gross total or subtotal);
- Had complete demographic, operative, and follow-up records available.

Patients were excluded if they:

- Had incomplete surgical data;
- Had surgery for a non-metastatic brain lesion;
- Underwent biopsy only or radiosurgery without open resection.

### 2.3. Data Collection and Variables

Clinical and demographic data were extracted from medical records, operative logs, and imaging systems. Collected variables included the following:

1. Sex, age, corrected age;
2. Number and location of brain metastases;
3. Eloquent zone involvement;
4. Primary tumour type and status (known/unknown at brain metastasis diagnosis);
5. Extracranial metastases (present or absent);
6. Karnofsky Performance Status (KPS) (>70 vs. ≤70);
7. Postoperative complications;
8. Comorbidity count and classification (categorised into 0 to 6+);
9. Recurrence count and time intervals;
10. Extent of resection (GTR vs. STR);
11. Postoperative mortality (in hospital).

### 2.4. Surgical Procedure and Classification

Based on preoperative imaging and patient condition, the neurosurgical team determined the surgical approach and extent of resection. The extent of resection was defined intraoperatively and confirmed radiologically by contrast-enhanced CT obtained within 24 h postoperatively. Early postoperative MRI was not available as part of routine practice during the study period. The extent of resection was defined as follows:

- Gross total resection (GTR): no residual enhancing tumour.
- Subtotal resection (STR): residual enhancing tumour present.

### 2.5. Postoperative Management

Patients were extubated in the operating room or transferred to and intubated in the neurosurgical ICU based on intraoperative stability. Neurological exams were conducted every 2 h during the first 24 h. Postop brain CT was obtained within 24 h to assess hematoma, infarction, or oedema. Patients received early mobilisation, thromboembolism prophylaxis, and pain control. Postoperative complications were recorded and graded according to the Clavien–Dindo classification system.

Outcome evaluation included the following:

- Postoperative complications (neurological or systemic within 30 days);
- Recurrence (local radiographic recurrence at the resection site);
- Functional status, measured using KPS, categorised as >70 vs. ≤70;
- Mortality;
- Overall survival (OS) was defined as the interval between the date of neurosurgical resection and the date of death from any cause or the last known follow-up for censored patients;
- Recurrence intervals (1–6, 7–12, 13–26, ≥27 months).

Gross total resection rate recurrence was defined as local radiographic recurrence at the resection cavity. For analysis, patients were further stratified according to the number of local recurrences observed during follow-up (single, multiple, or extensive).

### 2.6. Vital Status Confirmation

Survival status was confirmed using official correspondence from the Cluj County Directorate for Population Records (dated 27 May 2025), which provided verified dates of death or confirmed active status for long-term follow-up.

### 2.7. Statistical Analysis

All statistical analyses were performed using IBM SPSS Statistics v26.0 (IBM Corp., Armonk, NY, USA), with statistical significance set at *p* < 0.05. Continuous variables were summarised as mean ± standard deviation or median with interquartile range (IQR), while categorical variables were presented as frequencies and percentages. Mann–Whitney U tests were used to compare age across binary clinical outcomes. Chi-square tests assessed associations among categorical variables, including resection type, complications, recurrence, anatomical location, and functional status. Binary logistic regression models were utilized to identify independent predictors of six binary outcomes: postoperative complications, recurrence, KPS > 70, known primary tumour, eloquent zone involvement, and gross total resection (GTR), with covariates such as age, eloquence, complications, and comorbidity burden included as appropriate.

To address confounding by indication in GTR vs. STR comparisons, we estimated propensity scores (logistic regression) using age, sex, KPS > 70, eloquent zone, number of brain metastases, comorbidity count, known primary, and extracranial metastasis. We applied stabilized Inverse Probability of Treatment Weighting (IPTW) and assessed balance with standardized mean differences (target < 0.10). Outcomes (complications, recurrence, postoperative mortality) were modelled with binomial GLMs using IPTW and robust SEs.

### 2.8. Survival Analysis

Survival outcomes were assessed using Kaplan–Meier methodology. Patients alive at the last follow-up were right-censored. Subgroup analyses were performed by sex (male vs. female), extent of resection (gross total resection [GTR] vs. subtotal resection [STR]), and recurrence status (none, single, multiple, extensive). Survival distributions were compared using the log-rank test. Median survival times with 95% confidence intervals (CI) and mean survival estimates were reported.

This study was evaluated and granted ethical approval from the Ethics Committee of the University of Medicine and Pharmacy “Iuliu Hațieganu” Cluj-Napoca (Approval No: AVZ 231). Since this study was retrospective and used anonymised data, informed consent was waived.

## 3. Results

### 3.1. Patient Characteristics

Three hundred fifty-eight patients with brain metastases were included in the analysis. The median age was 65 years (IQR: 56–72), with a mean age of 63.1  ±  11.7 years. The age range was 18 to 86 years (Table 1 and Table 2).

The most common primary tumour was pulmonary (46.4%), followed by breast cancer (13.4%) and melanoma (12.8%). Brain metastases were solitary in 87.4% of patients and involved eloquent areas in 53.9%. The frontal lobe was the most commonly affected site (33.0%), followed by the parietal lobe (17.9%) and cerebellum (12.8%). Gross total resection was performed in 93.9% of patients, and postoperative complications occurred in 20.1%. Recurrence was recorded in 10.9% of patients, with most occurring within the first six months (6.4%). Nearly half of the cohort had no comorbidity (46.1%), while 53.9% had one or more comorbidities (Table 3, Table 4 and Table 5).

### 3.2. Age Comparisons Across Clinical Subgroups (Mann–Whitney U Tests)

Mann–Whitney U tests were used to find age variations across clinical outcomes. Patients with KPS > 70 were significantly older than those with KPS ≤ 70 (U = 13,379.5; *p* = 0.007). Similarly, younger patients were not significantly more likely to undergo gross total resection (U = 2870.5; *p* = 0.079), and younger age was also associated with no recurrence (U = 5270.5; *p* = 0.021). For age, no significant differences were observed with respect to postoperative complications (*p* = 0.290), eloquent zone involvement (*p* = 0.275), postoperative mortality (*p* = 0.284), or extracranial metastasis (*p* = 0.656). Age did not differ significantly based on the number of brain metastases (one vs. two or more; *p* = 0.714) or comorbidity status (none vs. any; *p* = 0.085), although the latter approached significance. Patients with gross total resection had no significant difference in the number of brain metastases compared to those with subtotal resection (U = 3799.0; *p* = 0.693). However, there were no significant differences in comorbidity count between patients undergoing gross total versus subtotal resection (U = 3856.0; *p* = 0.705), between patients with and without recurrence (U = 6761.5; *p* = 0.323), or between male and female patients in terms of the number of metastases (U = 14,634.0; *p* = 0.236).

### 3.3. Associations Among Categorical Variables (Chi-Square Test)

Male sex was significantly associated with unknown primary tumours (*p* = 0.002) but not with eloquent zone involvement, extracranial metastasis, KPS status, recurrence, complications, or comorbidity burden (*p* > 0.25). The number of brain metastases was significantly associated with both eloquent zone involvement (*p* = 0.001) and KPS > 70 (*p* = 0.0151). Metastasis interval categories also showed significant associations with eloquence (*p* = 0.001), known primary tumour (*p* = 0.043), and KPS (*p* = 0.015). Anatomical location was significantly associated with eloquent zone involvement (*p* < 0.001), KPS status (*p* < 0.001), and postoperative complications (*p* < 0.001) but not with extracranial spread, recurrence, or comorbidity burden.

Gross total resection was not significantly associated with eloquent zone involvement (*p* = 0.777), known primary tumours (*p* = 0.135), postoperative complications (*p* = 0.554), or recurrence (*p* = 0.526). However, it was significantly associated with comorbidity burden (*p* = 0.027), contrary to prior assumptions. Postoperative mortality was not significantly associated with recurrence count (*p* = 0.960), KPS, eloquence, comorbidities, or primary tumour status. Recurrence interval was also not significantly associated with extracranial spread (*p* = 0.954), but it was significantly linked to recurrence count (*p* < 0.001).

A Chi-square test evaluating the association between primary cancer type and survival status (alive vs. deceased) yielded a non-significant result (χ^2^ = 4.31, *p* = 0.366), indicating no strong association between cancer type and survival in this cohort. While subgroup comparisons were considered, the low number of deaths across cancer types limited interpretability. Additional data with more mortality variation would be required for meaningful pairwise analysis.

### 3.4. Additional Significant Associations

Sex was significantly associated with grouped pathology (*p* < 0.001) but not with the number of brain metastases (*p* = 0.081). The number of brain metastases showed a strong association with anatomical location (*p* < 0.001) but was not significantly related to metastasis interval (*p* = 0.980) or resection type (*p* = 0.798). Metastasis interval was also not associated with anatomical location (*p* = 1.000) or resection (*p* = 1). Anatomical location and resection demonstrated a significant relationship (*p* = 0.036). Eloquent zone involvement was significantly associated with KPS status (*p* < 0.001), postoperative complications (*p* < 0.001), and comorbidity burden (*p* = 0.008). Grouped pathology was not significantly associated with resection (*p* = 0.337). Primary tumour status was significantly associated with recurrence count (*p* = 0.008) but not with comorbidity burden (*p* = 0.090). KPS status was significantly linked to both postoperative complications (*p* < 0.001) and comorbidity burden (*p* < 0.001). A significant association was also found between complications and comorbidity burden (*p* = 0.002). However, the recurrence interval was not significantly associated with resection (*p* = 1).

### 3.5. Survival Analysis

**Figure 1:**
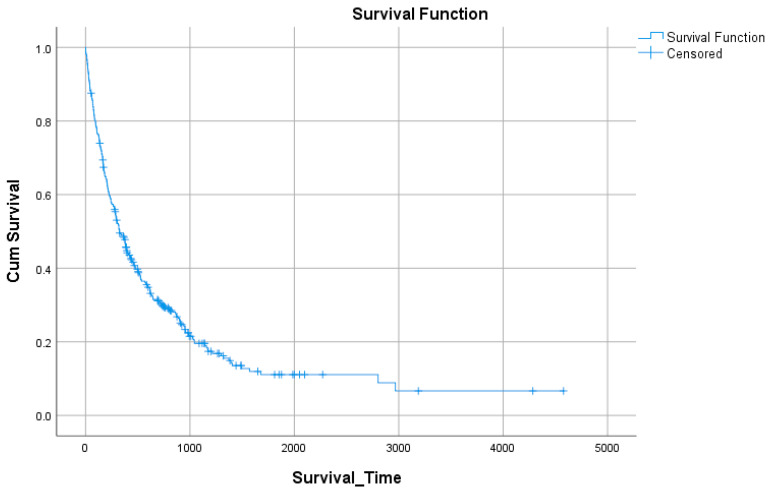
Overall survival curve (Kaplan–Meier).

Survival data were available for 354 patients, of whom 277 (78.2%) experienced the event of interest (death) and 77 (21.8%) were censored. The overall median survival time was 325 days (95% CI: 270–380), with a mean survival estimate of 812 days (95% CI: 645–980) (Figure 1, Table 6).

**Figure 2:**
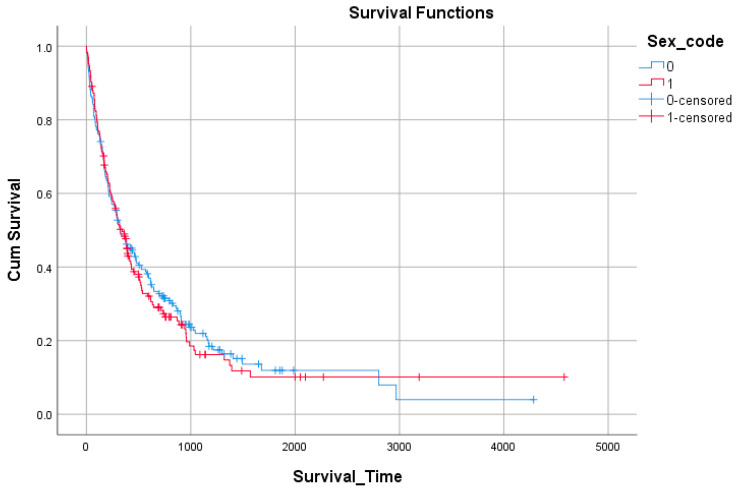
Survival by sex.

When stratified by sex, no significant difference was observed. Median survival was 324 days (95% CI: 225–423) for males and 343 days (95% CI: 265–421) for females, with overlapping confidence intervals (Figure 2, Table 7).

**Figure 3:**
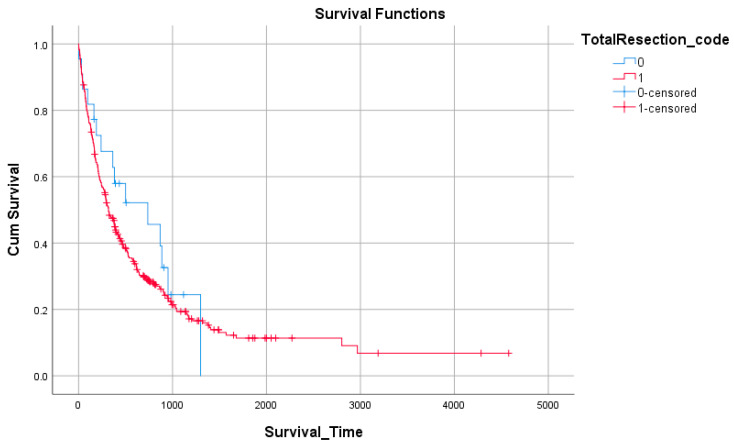
Survival by extent of resection.

Analysis by extent of resection demonstrated that patients undergoing subtotal resection (STR, n = 22) had a longer median survival (737 days, 95% CI: 157–1317) compared to those who underwent gross total resection (GTR, n = 332; 318 days, 95% CI: 262–374). However, interpretation is limited by the small number of STR cases (Figure 3, Table 8).

**Figure 4:**
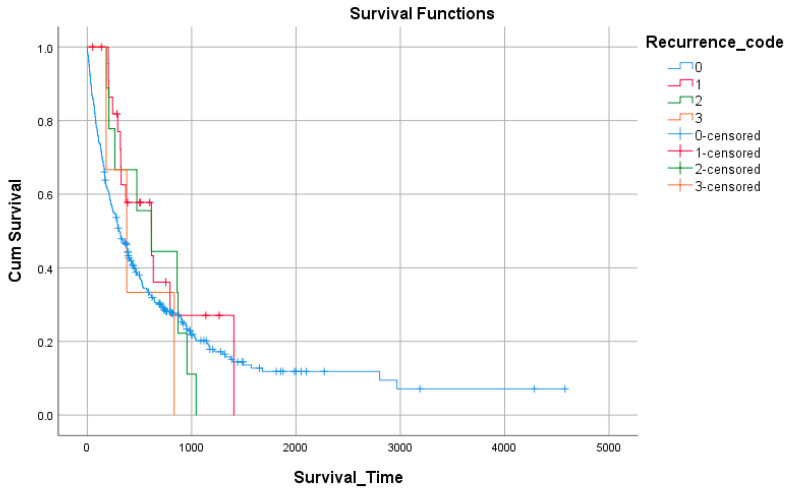
Survival by recurrence status.

Survival by recurrence status revealed variable outcomes. Patients without recurrence (n = 318) had a median survival of 302 days (95% CI: 232–372). Median survival appeared longer in those with recurrence—616 days for single recurrence (n = 24), 614 days for multiple recurrences (n = 9), and 378 days for extensive recurrence (n = 3)—although these subgroups were too small for definitive comparisons (Figure 4, Table 9).

Multivariable Survival Analysis (Cox regression)

We utilized a Cox proportional hazards model to assess predictors of overall survival.

Extent of resection. Patients undergoing gross total resection (GTR) had a significantly higher hazard of death compared with those undergoing partial/subtotal resection (HR = 1.44, 95% CI 1.08–1.93, *p* = 0.013).

Age. Each additional year of age was associated with an increased hazard of death (HR = 1.02 per year, 95% CI 1.01–1.03, *p* < 0.001).

Sex. Male sex was not significantly associated with overall survival (HR = 1.17, 95% CI 0.92–1.49, *p* = 0.212).

Older age and extent of resection are independent predictors of overall survival in this cohort, while sex did not significantly influence outcomes. The direction of association for GTR suggests that patients selected for more aggressive resection may have had worse underlying prognostic factors, contributing to higher observed hazard despite complete resection (Table 10).

### 3.6. Gross Total Resection and Mortality

A total of five patients died postoperatively. Among those who underwent GTR, mortality was 0.9% (3/336), compared to 9.1% (2/22) in patients with STR. This difference was statistically significant (Fisher’s exact test, *p* = 0.021), with STR associated with an approximately 11-fold increase in the odds of mortality. This finding suggests a potential survival benefit from gross total resection, although further studies with larger death counts are needed to confirm this association.

### 3.7. Binary Logistic Regression Analysis

We conducted multivariable binary logistic regression models to identify independent predictors of six key clinical outcomes: postoperative complications, recurrence, functional status (KPS > 70), whether the primary tumour was known, eloquent zone involvement, and gross total resection (GTR). Predictor variables included age, eloquent zone involvement, primary tumour status, postoperative complications, functional status, and extent of resection, depending on model context. In the postoperative complications model, higher functional status (KPS > 70) was strongly protective (OR = 0.05; *p* < 0.001), while other variables, including age, eloquence, primary tumour status, and GTR, were not significant. For recurrence, no predictors reached statistical significance. Although recurrence was less likely with increasing age (OR = 0.98; *p* = 0.082) and more likely in patients with known primaries (OR = 2.95; *p* = 0.009), neither GTR (OR = 2.39; *p* = 0.413) nor eloquence showed significant predictive value in the model.

In the model predicting functional independence (KPS > 70), both eloquent zone involvement (OR = 0.13; *p* < 0.001) and postoperative complications (OR = 0.06; *p* < 0.001) were independently associated with significantly lower odds of high KPS. Age was also a significant negative predictor (OR = 0.97; *p* = 0.022). GTR showed a non-significant trend toward better KPS (OR = 0.31; *p* = 0.056). In the model predicting whether the primary tumour was known, no variables were significantly associated. GTR (OR = 1.97; *p* = 0.136), KPS, eloquence, age, and complications all failed to reach significance. In the eloquent zone involvement model, only lower KPS remained a significant independent predictor (OR = 0.13; *p* < 0.001). GTR (OR = 0.55; *p* = 0.241), age, complications, and primary tumour status were not significant predictors.

Finally, in the model predicting gross total resection (GTR), higher KPS was the only significant predictor (OR = 0.30; *p* = 0.045). Contrary to expectations, neither eloquent location (OR = 0.56; *p* = 0.251), known primary tumour (OR = 2.02; *p* = 0.123), nor postoperative complications (OR = 0.43; *p* = 0.187) were significant predictors of GTR. These results diverge from clinical assumptions and highlight the complex interactions influencing surgical decisions.

## 4. Discussion

This retrospective review examined 358 patients who underwent resection of brain metastasis at a tertiary-level neurosurgical centre in South-Eastern Europe. It presents a detailed account of demographic, anatomical, and clinical features and characterises predictors of complications, recurrence, functional independence, and survival. Gross total resection (GTR) was achieved in 93.9% of patients (336 out of 358), while subtotal resection (STR) was not significantly associated with fewer complications (OR = 0.48, *p* = 0.244). Although GTR was not an independent predictor of functional independence or recurrence in multivariable analysis (OR = 2.39, *p* = 0.413), it was associated with significantly lower postoperative mortality (0.9% vs. 9.1% for STR, *p* = 0.021). Kaplan–Meier analysis of 354 patients demonstrated a median overall survival (OS) of 325 days, with no meaningful difference by sex and heterogeneous survival patterns across recurrence subgroups. Postoperative complications occurred in 20% of patients and were strongly associated with lower functional outcomes, underscoring the importance of perioperative care. Eloquent zone involvement remained a consistent predictor of poorer functional status, while advanced age was significantly associated with lower functional independence. Unknown primary tumour status was not significantly associated with any modelled outcomes. These findings emphasise the multifactorial nature of outcomes following surgery for brain metastasis, in which functional independence (as measured by postoperative KPS) and OS both emerge as key prognostic factors, and while comorbidity burden was not an independent predictor of GTR, systemic health may still influence surgical planning.

### 4.1. Overall Survival (OS)

Our observed overall survival of approximately 11 months is shorter than the 14–15 months reported in several prior studies. This discrepancy may in part reflect the lack of standardized data on adjuvant therapies and molecular profiling in our cohort, both of which are recognized determinants of survival. Prior studies, such as Liu et al. (2017) [10] and Kavouridis et al. (2019) [11], report median OS between 14 and 15.4 months. In our series, Kaplan–Meier analysis of 354 patients demonstrated a median OS of 325 days (95% CI: 270–380), broadly in line with these prior reports. Kavouridis et al. (2019) [11] similarly estimated OS at 15.4 months and validated the same prognostic factors; they also included molecular markers (EGFR, ALK) in their study, and we did not. Hulsbergen et al. (2022) [12] reported an overall survival (OS) of 14 months using machine learning models and found the same predictors, stressing that while the extent of resection did not independently predict OS in our cohort, it did influence perioperative mortality, underlining the need to separate short-term surgical risk from long-term oncologic outcomes.

Proescholdt et al. (2021) [13] reported that OS improvement and progression-free survival (PFS) were associated with gross total resection (GTR) and having solitary lesions. While GTR was frequently performed, it was not significantly associated with recurrence or complications in our cohort. However, in our study, GTR was significantly associated with lower postoperative mortality (0.9% vs. 9.1% for STR, *p* = 0.021), suggesting a potential survival benefit.

Yardeni et al. (1984) [14] reported an OS of 6.6 months with a 15% mortality rate, possibly due to the limited technique back then. Nonetheless, their original finding that lesion location impacts prognosis matched our conclusions. Similarly, Tabouret et al. (2012) [15] suggested improved survival is due to multimodal management, which echoes our determination of OS as 14 months and treatment strategy. Dasgupta et al. (2021) [16] reported OS of 106 months, likely attributable to modern targeted therapies, and, as with prior reports, this reinforces the importance of tumour biology and baseline functional status in shaping survival, often more so than surgical extent alone. Michel et al. (2022) [17] reported HER2 status, long disease-free interval, and KPS ≥ 90% as prognostic factors associated with OS ≥ 3 years, thus consistent with our emphasis on KPS and solitary lesions. Long-term survivors had an OS of 51.5 months (Hüghel et al. 2023) [18] relating to oligo-metastatic disease and high KPS (also aligning with our results). The same study also established that GTR and non-eloquent locations were favourable predictors, aligning with Ene and Ferguson 2022 [19]. Xu et al. (2022) [20] reported an OS of 26.7 months in NSCLC patients with solitary lesions, which could be related to stricter inclusion criteria and more aggressive adjuvant therapy to which patients were subjected. Suh et al. (2020) [21] validated the survival benefit of multimodal treatment and made associations for KPS, lesion number, and extracranial spread. Cuthbert et al. (2023) [22] presented a lower OS (8.1 months), perhaps related to the different therapies used, but did identify the same prognosis-related predictors. Arita et al. (2025) [23] found improved OS (21.8 months) with salvage surgery in contrast to our findings that GTR conferred no OS advantage over STR; nevertheless, Jędrys et al. (2023) [24] found poor OS (~12 months) in patients with sarcoma metastases, further highlighting tumour biology as a consideration. Gusho et al. (2021) [9] reported even shorter survival (DFS 6 months), consistent with the high systemic disease burden in their cohort.

### 4.2. Recurrence

While multiple prior studies have reported a decreased incidence of recurrence following gross total resection (GTR), we did not observe a significant protective effect of GTR on recurrence in our cohort. GTR was not significantly associated with recurrence (OR = 2.39, *p* = 0.413). Proescholdt et al. (2021) [13] demonstrated that GTR was associated with lower recurrence and complication rates, as well as improved functional outcomes, aligning with the traditional understanding that more extensive resection improves local control. Liu et al. (2019) [25] showed that surgery combined with whole-brain radiotherapy (WBRT) resulted in lower recurrence rates than surgery alone, reinforcing the benefit of multimodal treatment. Arita et al. (2025) [23] found that GTR in salvage cases did not confer a survival advantage over subtotal resection (STR), though patients with radiation necrosis in that study achieved a mean survival of 68.5 months. Jędrys et al. (2023) [24] reported particularly high recurrence rates in patients with sarcoma brain metastases, even with multimodal therapy, highlighting the dominant role of tumour biology in recurrence risk over the extent of surgical resection alone.

### 4.3. Complications and Surgical Risk

Extremely low surgical mortality (1.4%) demonstrates the potential of modern perioperative developments. Although Yardeni et al. (1984) [14] reported 15% mortality outlining the historical difference, they also noted that the location of the lesion plays a significant role in outcome. Proescholdt et al. (2021) [13] found that GTR was associated with fewer postoperative deficits and improved outcomes. In our cohort, GTR was not significantly associated with fewer complications, though it did not independently predict better functional status. Ene and Ferguson (2022) [19] also reported a higher risk in eloquent areas. Similarly to Ene and Ferguson (2022), we report fewer resections and worse outcomes in eloquent regions [19].

Gusho et al. (2021) [9] reported that sarcoma BMs had poor postoperative outcomes related to systemic disease burden. This explains the significantly different safety profile of surgery reported in our cohort compared to theirs. Arita et al. (2025) [23] demonstrated that salvage surgery may still be safely performed in patients with a history of prior radiotherapy, supporting selective surgical management. The consistent risk factors across all studies associated with eloquence, initial radiation, and systemic disease are consistent with our findings.

### 4.4. Prognostic Factors

Factors associated with clinical outcomes in our analysis included high KPS, younger age, and eloquent zone involvement. KPS is the most consistently validated prognostic factor. Liu et al. (2017) [10], Hügel et al. (2023) [18], Michel et al. (2022) [17], and Cuthbert et al. (2023) [22] have all confirmed that KPS has prognostic value for overall survival. Patients with higher KPS in these studies had better OS and PFS. Proescholdt et al. (2021) [13], Dasgupta et al. (2021) [16], and Michel et al. (2022) [17] observed that solitary lesions led to increased OS and decreased rates of recurrence. In contrast, Ene and Ferguson (2022) [19] noted increased QoL associated with solitary lesions. Our findings regarding predictors of recurrence and complications were consistent with these reported OS outcomes in the literature. Liu et al. (2017) [10], Gusho et al. (2021) [9], and Arita et al. (2025) [23] confirmed that extracranial disease was associated with worse outcomes, and in our analysis. Regarding age, patients 65 years old or older were associated with worse OS in the literature (Michel et al. (2022) [17], Pojskic et al. (2017) [26]). Regarding GTR, previous studies [10,13,19] found a positive association with OS; however, in our data, GTR was not significantly associated with recurrence. The observed non-significant trend toward higher recurrence in the GTR group may reflect tumour biology or selection bias, rather than a true causal effect. Eloquent region involvement was not associated with reduced resectability (*p* = 0.251) and poorer outcomes in our study and in other studies [19], emphasizing that this requires careful planning. Michel et al. (2022) [17] created a prognostic score (AUC > 0.77) and Hulsbergen et al. (2022) [12] developed an ML model, both using KPS, age, lesion location, and treatment as variables, consistent with our findings. Molecular markers (Kavouridis et al. (2019) [11], Xu et al. (2022) [20], and Suh et al. (2020) [21]) had mixed results using EGFR, ALK, etc. We did not include molecular markers in our analysis, but future studies should consider these variables.

### 4.5. Adjunct Therapies and Multimodal Management

Our multimodal treatment approach, which included surgery, WBRT/SRS, and systemic therapy when indicated, is consistent with strategies shown to improve OS and PFS in prior studies. Liu et al. confirmed in 2019 [25] that WBRT following surgery improved outcomes. Xu et al. (2022) [20] confirmed that WBRT, chemotherapy, and targeted therapy were strong independent predictors of OS. Suh et al. (2020) [21] emphasised the importance of stratifying multimodal care, a principle reflected in our treatment approach. Furthermore, Tabouret et al. (2012) [15] suggested that improved survival highlights an ‘intensified treatment’ occurring, reflecting our practice. Arita et al. (2025) [23] found that salvage surgery could result in improved survival following radiotherapy, including in cases of necrosis. Michel et al. (2022) [17] indicated that long metastasis-free intervals were predictors of survival and suggested that improved survival with multimodal care might be observed in patients with slower tumour biology. Dasgupta et al. (2021) [16] observed long-term OS improvements in patients with local therapy and where targeted therapy was an option. Cuthbert et al. (2023) [22] had lower OS due to inconsistency in the systemic therapy and provided further support for systematic multimodal therapies. Hulsbergen et al. (2022) [12] examined the incorporation of SRS and immunotherapy in ML-based survival modelling, highlighting the changing standards of care. Suh et al. (2020) [21] and Sacks and Rahman (2020) [1] both identified GPA-model-based, individualised multimodal strategies, which fit entirely with our current work.

### 4.6. Particularities of the Study

The strength of our study is the large, well-characterised cohort of 358 patients who were surgically managed for brain metastases, as well as from a South-Eastern European neurosurgical centre, which is underrepresented in the literature. We collected extensive data on essential variables, which allowed for detailed multivariate analyses of predictors of functional outcome, recurrence, and overall survival. However, the retrospective design does introduce the possibility of selection and information biases, and the absence of molecular profiling limits the relevance of our findings in precision oncology. An important limitation of this study is the lack of follow-up data regarding patient response to adjuvant therapies such as chemotherapy and radiotherapy, as these treatments were administered locally in each patient’s county and not controlled or monitored within our institution. Although earlier exploratory analysis suggested an association between primary cancer type and survival, formal testing showed no statistically significant link (*p* = 0.366), and post hoc interpretation was limited due to insufficient mortality variation across pathology groups. Even so, the results provide valuable insights that can support surgical planning, guide multidisciplinary discussions, and assist in patient counselling. Future prospective studies should explore the impact of molecular profiling, comprehensive systemic therapy data, and patient-reported quality-of-life outcomes, especially in patients with multiple metastases or tumours located in eloquent brain regions. This will help optimise truly personalised treatment approaches. Our study is limited by the lack of systematic data on adjuvant therapies and molecular profiling, which may influence outcomes. Despite this, the large cohort highlights KPS as a key predictor of surgical risk, and future studies incorporating treatment and molecular data are needed for more precise prognostication. Despite IPTW, residual confounding and small-event bias (five deaths) limit causal inference; thus GTR–STR comparisons should be interpreted cautiously. Because KPS was only available in categorical form (≤70 vs. >70) in our institutional database, we were unable to model it as a continuous or ordinal predictor, which may have reduced sensitivity to detect nuanced prognostic effects. The STR subgroup was very small (n = 22), and residual confounding by indication likely persists despite adjustments, limiting causal inference for STR–GTR survival comparisons.

## 5. Conclusions

This retrospective cohort study demonstrates that gross total resection (GTR) was not independently associated with lower postoperative complication rates or reduced recurrence. The observed non-significant trend toward higher recurrence in the GTR group may reflect selection bias or tumour biology. In contrast, functional status, eloquent zone involvement, and age were independently correlated with postoperative outcomes. Although GTR appeared associated with lower postoperative mortality, this finding was based on very few deaths and should be interpreted with caution. Median overall survival (OS) was 325 days, providing a reference point for long-term outcomes in this regional cohort. These findings underscore the importance of individualized surgical planning and support future research integrating molecular and systemic treatment variables to guide personalized neuro-oncologic care.

## Figures and Tables

**Table 1:** Age distribution by sex.

Sex	N	Mean ± SD	Median (IQR)
**Male**	191	65.8 ± 10.7	69 (59–73)
**Female**	167	60.1 ± 12.0	61 (52–69)

**Table 2:** Patients’ characteristics.

Category	Characteristic	N	%
**Sex**	Male	191	53.4%
	Female	167	46.6%
**KPS**	KPS > 70	175	48.9%
	KPS < 70	183	51.1%
**Tumour Characteristics**	Primary tumour known	209	58.4%
	Pulmonary primary	166	46.4%
	Breast cancer	48	13.4%
	Melanoma	46	12.8%
	Clear cell Renal cell carcinoma	36	10.1%
	Other	62	17.3%
**Brain Metastasis**	Single brain metastasis	313	87.4%
	Multiple metastases	45	12.6%
	Eloquent zone involvement	193	53.9%
	Non-eloquent zone	165	46.1%
	Frontal	119	33.2%
	Parietal	64	17.9%
	Cerebellum	46	12.8%
	Temporal	35	9.8%
	Multiple locations	45	12.6%
	Occipital	26	7.3%
	Brainstem	8	2.2%
	Sellar region	4	1.1%
	Insular	4	1.1%
	Basal ganglia	4	1.1%
	Thalamus	3	0.8%
**Clinical Course**	Gross Total Resection	336	93.9%
	Subtotal Resection	22	6.1%
**Complications**	Postop complications	72	20.1%
	No complications	286	79.9%
**Mortality**	Mortality	5	1.4%
	No mortality	353	98.6%
**Recurrence**	Recurrence	39	10.9%
	No recurrence	319	89.1%
**Comorbidities**	No comorbidity	165	46.1%
	1–6 comorbidities	193	53.9%
**Recurrence**	1–6 months	23	6.4%
	7–12 months	7	2.0%
	13–26 months	5	1.4%
	<27 months	1	0.3%

**Table 3:** IPTW treatment effect (GTR vs. STR): weighted odds ratios.

Outcome	IPTW OR	95% CI Lower	95% CI Upper	*p*-Value
Postop complications	0.59	0.17	2.03	0.402
Recurrence	1.29	0.39	4.24	0.678
Postop mortality	0.15	0.02	1.14	0.067

**Table 4:** Crude treatment effect: odds ratios.

Outcome	Crude OR	95% CI Lower	95% CI Upper	*p*-Value
Postop complications	0.68	0.32	1.45	0.317
Recurrence	1.45	0.62	3.40	0.393
Postop mortality	0.09	0.02	0.51	0.006

**Table 5:** Baseline characteristics of patients by extent of resection.

Variable	GTR (n = 336)	STR (n = 22)	*p*-Value
Age (years, median [IQR])	65.0 (55.8–71.0)	68.5 (61.5–75.0)	0.079
Male sex (%)	53.0	59.1	0.737
KPS > 70 (%)	48.2	59.1	0.442
Eloquent location (%)	53.6	59.1	0.778
Number of brain metastases (median [IQR])	1.0 (1.0–1.0)	1.0 (1.0–1.0)	0.693
Comorbidity count (median [IQR])	1.0 (0.0–2.0)	1.0 (0.0–2.0)	0.845
Primary tumour known (%)	58.0	68.2	0.337
Extracranial metastases (%)	15.2	18.2	0.754

**Table 6:** Overall survival—number at risk.

Time (Days)	All Patients
0	354
180 (6 mo)	231
365 (12 mo)	164
730 (24 mo)	89
1095 (36 mo)	43

**Table 7:** Survival by sex—number at risk.

Time (Days)	Male	Female
0	191	163
180 (6 mo)	119	112
365 (12 mo)	81	83
730 (24 mo)	42	47
1095 (36 mo)	22	21

**Table 8:** Survival by extent of resection—number at risk.

Time (Days)	GTR	STR
0	332	22
180 (6 mo)	216	15
365 (12 mo)	152	12
730 (24 mo)	80	9
1095 (36 mo)	39	4

**Table 9:** Survival by recurrence status—number at risk.

Time (Days)	No Recurrence	Once	Twice	3 Times	5 Times
0	316	26	9	3	0
180 (6 mo)	195	24	9	3	0
365 (12 mo)	142	14	6	2	0
730 (24 mo)	74	5	4	1	0
1095 (36 mo)	36	3	0	0	0

**Table 10:** Multivariable Cox proportional hazards model for overall survival.

Variable	HR (95% CI)	*p*-Value
Extent of resection	1.44 (1.08–1.93)	0.013
Age at surgery (years)	1.02 (1.01–1.03)	<0.001
Sex (male vs. female)	1.17 (0.92–1.49)	0.212

Coded 1 = GTR, 0 = partial/STR (HR > 1 indicates higher hazard for GTR vs. partial/STR).

## Data Availability

All data are available from the corresponding author upon reasonable request. The anonymized dataset analysed in this study is part of a secure institutional registry, and access requires prior approval from the ethics committee. De-identified data may be shared with qualified researchers upon request and subject to ethics approval. The statistical analysis code is available from the corresponding author.

## Abbreviations

The following abbreviations are used in this manuscript:

Abbreviation	Full Term
BM	Brain Metastasis
GTR	Gross Total Resection
STR	Subtotal Resection
KPS	Karnofsky Performance Status
WBRT	Whole-Brain Radiotherapy
SRS	Stereotactic Radiosurgery
OS	Overall Survival
PFS	Progression-Free Survival
OR	Odds Ratio
CI	Confidence Interval
MRI	Magnetic Resonance Imaging
ICU	Intensive Care Unit
CT	Computed Tomography
